# Cladosporol A triggers apoptosis sensitivity by ROS-mediated autophagic flux in human breast cancer cells

**DOI:** 10.1186/s12860-017-0141-0

**Published:** 2017-07-20

**Authors:** Mytre Koul, Ashok Kumar, Ramesh Deshidi, Vishal Sharma, Rachna D. Singh, Jasvinder Singh, Parduman Raj Sharma, Bhawal Ali Shah, Sundeep Jaglan, Shashank Singh

**Affiliations:** 1 Cancer Pharmacology Division, CSIR-Indian Institute of Integrative Medicine, Jammu, India; 2 Natural Product Chemistry, CSIR-Indian Institute of Integrative Medicine, Jammu, India; 3 Microbial Biotechnology Division, CSIR-Indian Institute of Integrative Medicine, Jammu, India; 4 Academy of Scientific & Innovative Research (AcSIR), CSIR, New Delhi, India; 5 Department of Conservative Dentistry & Endodontics, Indira Gandhi Govt. Dental College and Hospital, Jammu, India

**Keywords:** Apoptosis, Breast cancer, *Cladosporium cladosporioides*, Cladosporol a, Endophytes, Reactive oxygen species

## Abstract

**Background:**

Endophytes have proven to be an invaluable resource of chemically diverse secondary metabolites that act as excellent lead compounds for anticancer drug discovery. Here we report the promising cytotoxic effects of Cladosporol A (HPLC purified >98%) isolated from endophytic fungus *Cladosporium cladosporioides* collected from *Datura innoxia*. Cladosporol A was subjected to in vitro cytotoxicity assay against NCI60 panel of human cancer cells using MTT assay. We further investigated the molecular mechanism(s) of Cladosporol A induced cell death in human breast (MCF-7) cancer cells. Mechanistically early events of cell death were studied using DAPI, Annexin V-FITC staining assay. Furthermore, immunofluorescence studies were carried to see the involvement of intrinsic pathway leading to mitochondrial dysfunction, cytochrome c release, Bax/Bcl-2 regulation and flowcytometrically measured membrane potential loss of mitochondria in human breast (MCF-7) cancer cells after Cladosporol A treatment. The interplay between apoptosis and autophagy was studied by microtubule dynamics, expression of pro-apoptotic protein p21 and autophagic markers monodansylcadaverine staining and LC3b expression.

**Results:**

Among NCI60 human cancer cell line panel Cladosporol A showed least IC_50_ value against human breast (MCF-7) cancer cells. The early events of apoptosis were characterized by phosphatidylserine exposure. It disrupts microtubule dynamics and also induces expression of pro-apoptotic protein p21. Moreover treatment of Cladosporol A significantly induced MMP loss, release of cytochrome c, Bcl-2 down regulation, Bax upregulation as well as increased monodansylcadaverine (MDC) staining and leads to LC3-I to LC3-II conversion.

**Conclusion:**

Our experimental data suggests that Cladosporol A depolymerize microtubules, sensitize programmed cell death via ROS mediated autophagic flux leading to mitophagic cell death.

**Graphical abstract:**

The proposed mechanism of Cladosporol A -triggered apoptotic as well as autophagic death of human breast cancer (MCF-7) cells. The figure shows that Cladosporol A induced apoptosis through ROS mediated mitochondrial pathway and increased p21 protein expression in MCF-7 cells *in vitro.* 
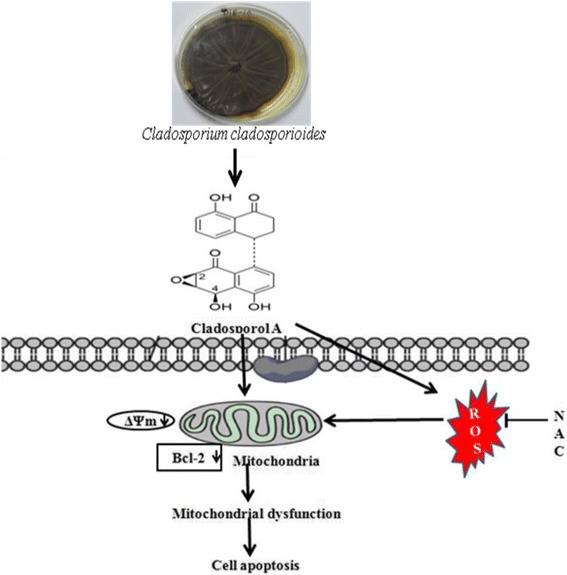

**Electronic supplementary material:**

The online version of this article (doi:10.1186/s12860-017-0141-0) contains supplementary material, which is available to authorized users.

## Background

Natural products from microbial source produce a vast wealth of specialized metabolites with wide range of structural diversity and biological activities. Secondary metabolites from fungal endophytes and their synthetic analogues have been widely used in pharmaceutical industry. In the past two decades variety of promising bioactive compounds have been successfully discovered from fungal endophytes having insecticidal, antimicrobial and anticancer activities [1]. It has been estimated that, in last 50 years about 47% of the anticancer drugs have been obtained either as natural products or synthetic compounds derived from them [2]. Especially fungal endophytes have produced a plethora of bioactive compounds having significant cytotoxic potential e.g. Paclitaxel, Podophyllotoxin, Vinblastine, Vincristine, Camptothecine, Mycophenolic acid, Emodin, Wortmannin, Dicatenarin etc. [3–5]. Among various fungal endophytes, *Cladosporium* species are also well known to produce variety of secondary metabolites which find promising application in pharmaceutical industry [6]. In regard to their anticancer potential, Earlier in 2007 Wang et al. isolated endophytic *Cladosporium Sp* from *Quercus variabilis.* They further purified and characterized a unique fungal metabolite Brefeldin A from crude extract of *Cladosporium sp.* [7]. Brefeldin A exhibited significant antitumor activity, its treatment causes apoptosis in several human cancer cell lines e.g. human glioblastoma (SA4, SA146, U87MG) and colon cancer cells (HCT-116) via arresting G_0_/G1 phase of cell cycle independent of Bcl-2/ Bax Mcl-1 and p53 [5]*.* In 2009 Zang and collegues, isolated Taxol from *Cladosporium cladosporioides* [8]. In the present study we isolated an endophytic fungus from *Datura innoxia* a well-known Indian annual medicinal plant. It belongs to the family Solanaceae [9]. *Datura innoxia* has been widely used as a traditional medicine in ayurveda since long times due to its immense medicinal properties, as all parts of the plants i.e. flowers, leaves, seed, root have appropriate medicinal applications. Its medicinal properties are due to the presence of about more than 30 alkaloids including atropine, hyoscyamine, scopolamine, withanolides (lactones) and other tropanes as well [10]. The methanolic leaf extract of *Datura innoxia* has shown to induce apoptosis in human colon adenocarcinoma (HCT 15) and larynx (Hep-2) cancer cell lines via inhibiting the expression of antiapoptotic Bcl-2 protein [11]. In view of its (*Datura innoxia*), promising cyotoxic effects, we isolated an endophytic fungus *Cladosporium cladosporioides* from it*.* We further isolated, purified and characterized a secondary metabolite Cladosporol A from endophytic *Cladosporium cladosporioides* and investigated the cyotoxic effects of Cladosporol A treatment against various human cancer cell lines. It exhibited promising cytotoxic effect against human breast (MCF-7) cancer cell line having minimum IC_50_ 8.7 μM. We next, ascertained mechanistically the cell death caused by Cladosporol A against breast cancer (MCF-7) cells. Breast cancer represents the second leading cancer in women worldwide. It is molecularly and clinically heterogeneous disease representing about 25% of all cancers in women and 12% of all new cancer cases [12]. It usually occurs in the breast tissue; starting in the lobules or ducts. The two major routes of cell death i.e. apoptosis and autophagy are highly controlled and dynamic processess that are used to remove damaged and defective cells. Upregulation of mitochondrial apoptosis pathway in response to antitumor agents is considered a signature of intrinsic apoptosis pathway in tumor cell lines. Apoptotic signals that trigger activation of mitochondrial pathway will result in MMP loss and cytochrome c release in mitochondrial inter- membrane space [4]. Autophagy, is a complex process which involves sequestration of intracellular organelles and cytoplasmatic portions into vacuoles called autophagosomes which further fuse with lysosomes to generate autophagolysosomes and mature lysosomes, where the whole material is degraded ultimately leading to cell death [13]. In addition, redox status of the cell i.e. reactive oxygen species (ROS) generation is a determining factor in regulating cell death pathways [14]. Here we first time report the involvement of ROS generation as major features of the apoptotic cell death caused by Cladosporol A in human breast (MCF-7) cancer cell line. Cladosporol A treatment induces membrane potential loss of mitochondria, cytochrome c release, Bax upregulation and Bcl-2 down regulation, thereby inducing mitochondrial activation mediated apoptosis. Cladosporol A also inhibited the assembiling of microtubules and induction of p21 a pro-apoptotic protein. Furthermore, Cladosporol A treatment also induced mild autophagic flux in human breast (MCF-7) cell line. Collectively the data, suggest that Cladosporol A, a microtubule de-polymerizer triggers mitochondrial cell death machinery and could be used as potential chemotherapeutic agent against human breast cancer.

## Results

### Identification, characterization and phylogenetic analysis of endophytic fungus (MRCJ-314) revealed it as *Cladosporium cladosporioides*

**Fig. 1:**
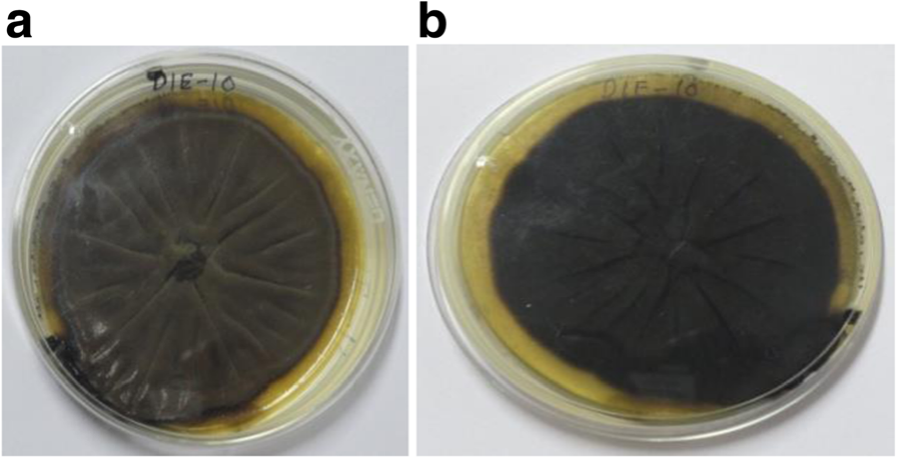
Morphology of isolate MRCJ-314 (*Cladosporium cladosporioides*); Appearance of colonies on PDA plate. The obverse (**a**) and reverse (**b**) view

The morphological characteristics of isolated endophytic fungus from *Datura innoxia* MRCJ-314 (DIE-10) supports that it belongs to genus *Cladosporium* [15]. Morphologically, in obverse view on PDA (potato dextrose agar plate), MRCJ-314 (DIE-10) showed dark olive green growth, velvety and on reverse view it seems olivaceous black (Fig. 1).

**Fig. 2:**
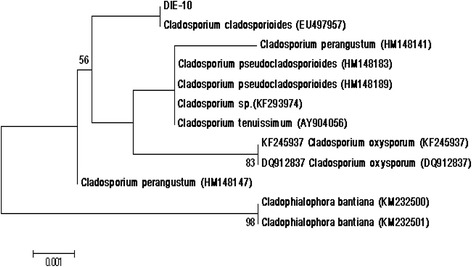
Neighbor-Joining tree of fungal endophyte MRCJ-314 (*Cladosporium cladosporioides*) based on ITS1–5.8S–ITS2 rDNA sequences. Confidence values above 50% obtained from a 1000-replicate bootstrap analysis are shown at the branch nodes

To designate MRCJ-314 (DIE-10) taxonomically up to species level, molecular technique was employed. The NCBI GenBank search for DNA sequence similarity showed that ITS1–5.8S–ITS2 sequence of DIE-10 has 99% homology with *Cladosporium cladosporioides* (GenBank Accession No. EU497597). Sequences of the maximum identity greater than 90% were retrieved, aligned with the sequence of strain MRCJ-314 (DIE-10), using clustal W module of MEGA6 software further subjected to neighbor-joining (NJ) analysis to obtain the phenogram (Fig. 2). The ITS sequence of strain (MRCJ-314) DIE-10 has highest nucleotide similarities with *Cladosporium cladosporioides* (EU497597), formed a clade with 100% bootstrap support indicating MRCJ-314 (DIE-10) as *C. cladosporioides.*

### Cladosporol A treatment effectively inhibited proliferation of human breast cancer (MCF-7) cell as well as their colony formation capacity

**Fig. 3:**
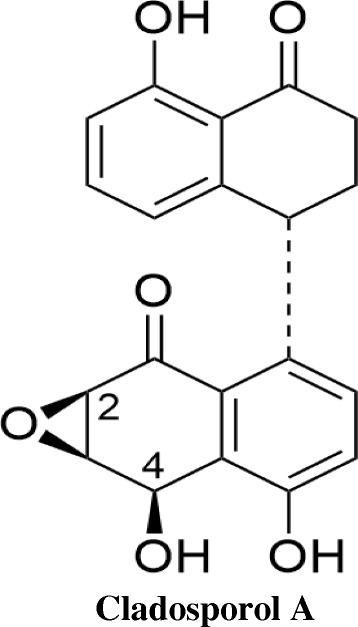
Structure of Cladosporol A

**Fig. 4:**
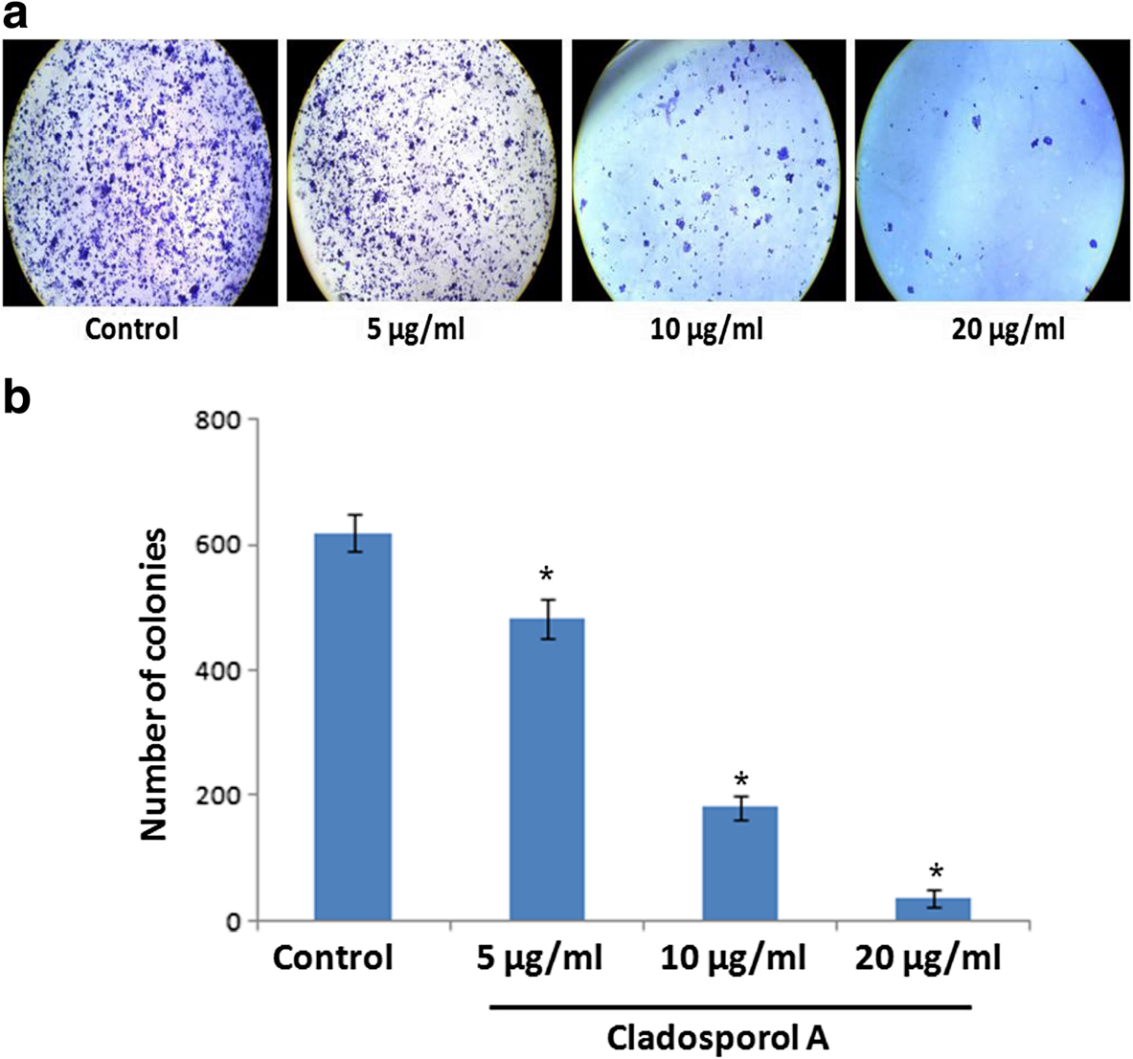
Clonogenic ability was assessed in human breast cancer (MCF-7) cells, after Cladosporol A treatment. The representative images are shown. **a** Reduction in colony forming capability was determined in MCF-7 cells. Cells (1 × 10^3^/ml/well) were seeded in six well plates and treated with different concentrations of Cladosporol A (5, 10, 20 μM). The number of crystal violet stained colony were counted randomly after seven days, quantified and photographed. **b** Data are presented as mean ± S.D., statistical analysis was done with **p < 0.01 and ***p < 0.001

Antiproliferative effect of Cladosporol A (Fig. 3) was demonstrated against human cancer cell line, panel via MTT assay. Cladosporol A treatment significantly inhibited cell proliferation of human breast cancer (MCF-7) cells in concentration-dependent manner with least IC_50_ 8.7 μM after 48 h (Table 1). Furthermore, on determining the ability of human breast (MCF-7) cancer cells to form colonies after Cladosporol A treatment revealed that it causes reduction in both the number as well as size of MCF-7 colonies as compared to untreated cells thereby suppressing ability of MCF-7 cells to form colonies in a concentration dependent manner (Fig. 4).

**Table 1:** IC_50_ values of Cladosporol A against NC1 60 panel of human cancer cell lines of different tissue origin. Data are expressed as the mean ± SD of three similar experiments

IC_50_(μM)					
S.No	MCF-7 (Breast cancer)	A549 (Lung cancer)	HCT-116 (Colon cancer)	PC-3 (Prostate cancer)	OVCAR-3 (Ovarian Cancer)
Cladosporol A	8.7 ± 0.205	11.7 ± 0.505	12 ± 0.505	15.6 ± 0.360	10.3 ± 0.556
Paclitaxel	0.0.25 ± 0.001	0.75 ± 0.05	8.5 ± 0.620	0.48 ± 0.003	2.6 ± 0.120
Doxorubicin	0.1 ± 0.006	0.087 ± 0.009	0.096 ± 0.005	0.05 ± 0.006	0.13 ± 0.008

### Cladosporol A treatment causes G1 phase arrest, depolymerizes microtubules and increases the expression of protein p21 of human breast cancer (MCF-7) cells

**Fig. 5:**
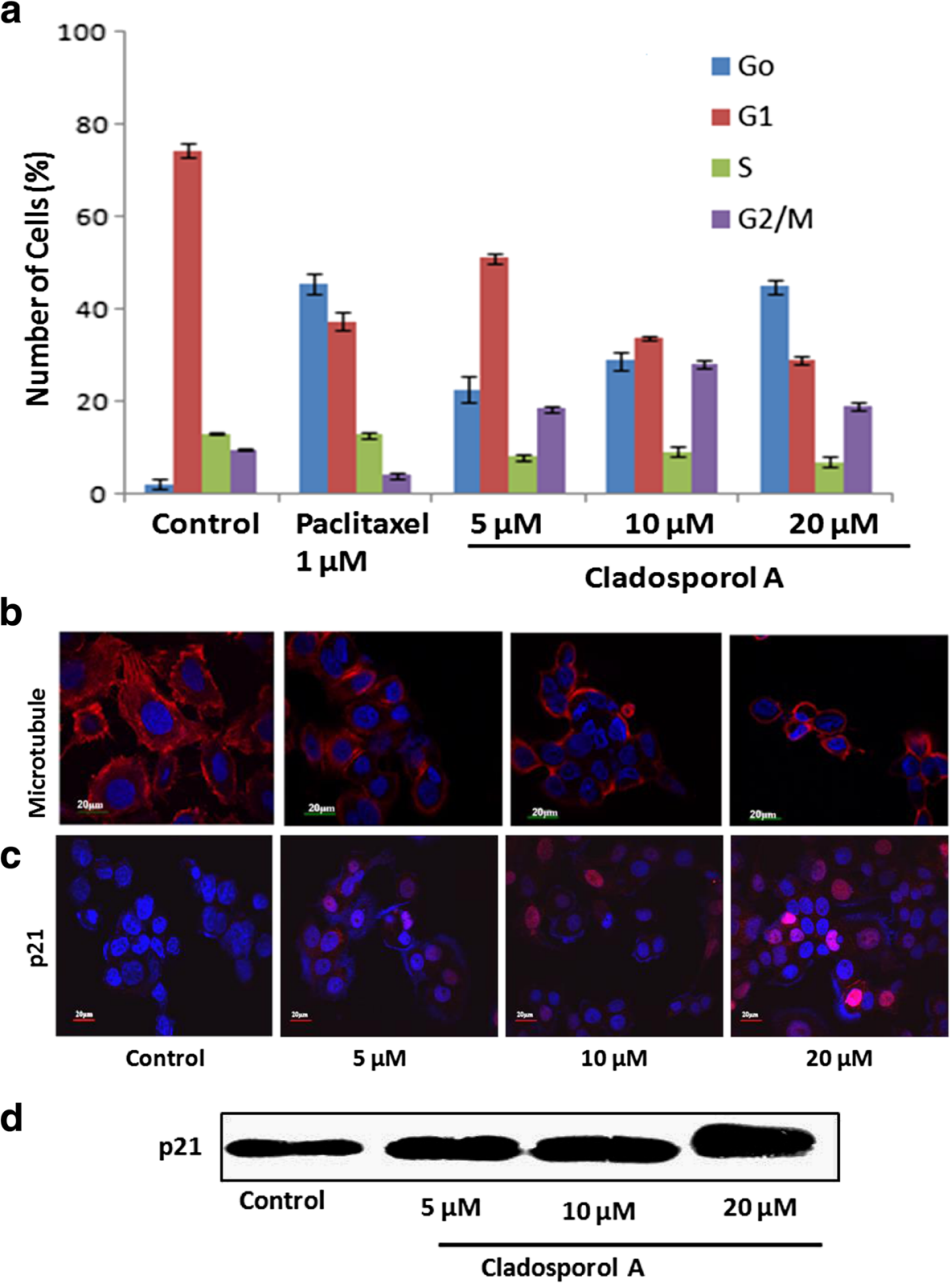
**a** Cell cycle distribution in human breast cancer (MCF-7) cells treated with 5, 10, 20 μM of Cladosporol A. Data are expressed as the mean ± SD of three similar experiments. **b** Cladosporol A disrupts the microtubules of human breast cancer (MCF-7) cells. Cells were treated with indicated concentrations of Cladosporol A for 24 h. Immunocytochemical staining was conducted using anti α-tubulin antibody and Alexa Flour-555-labelled secondary antibody and nuclei were stained with DAPI (**c**). Analysis of p21 protein expression of human breast cancer (MCF-7) cells after 24 h of Cladosporol A (5, 10, 20 μM) treatment by immunofluorescence microscopy (**d**). Western blot indicating the increase in p21 protein expression after Cladosporol A treatment in concentration dependent manner

The cell cycle distribution of Cladosporol A treated human breast cancer (MCF-7) cells was observed via flow-cytometry. After 24 h treatment with different concentrations of Cladosporol A, it causes G0/G1 phase arrest at higher concentration and accumulation of some population of cells in the G2/M phase at lower concentration (Fig. 5a). The percentage of G0 phase increased up to a high level at 20 μM (45%). To mechanistically investigate the basis of growth inhibitory effects of Cladosporol A, we next elucidated its effect on the expression of key proteins involved in cell proliferation by immunofluorescence microscopic studies. We investigated the effect of Cladosporol A on microtubules by immunofluorescence microscopy. Our data elucidated a concentration-dependent reduction in microtubule density after 24 h Cladosporol A treatment whereas control cells showed a typical array of radial, interphase microtubules (Fig. 5b). Thus indicating that Cladosporol A depolymerizes microtubules in MCF-7 cells. Further, we analysed the effect of Cladosporol A treatment on expression of cyclin-dependent kinase inhibitor p21 via immunofluorescence microscopic studies as well as western blot analysis. Our data suggested a concentration-dependent increase in expression of cyclin-dependent kinase inhibitor p21 (Fig. 5c and d).

### Cladosporol A induced significant chromatin condensation and triggers apoptosis in human breast cancer (MCF-7) cells

**Fig. 6:**
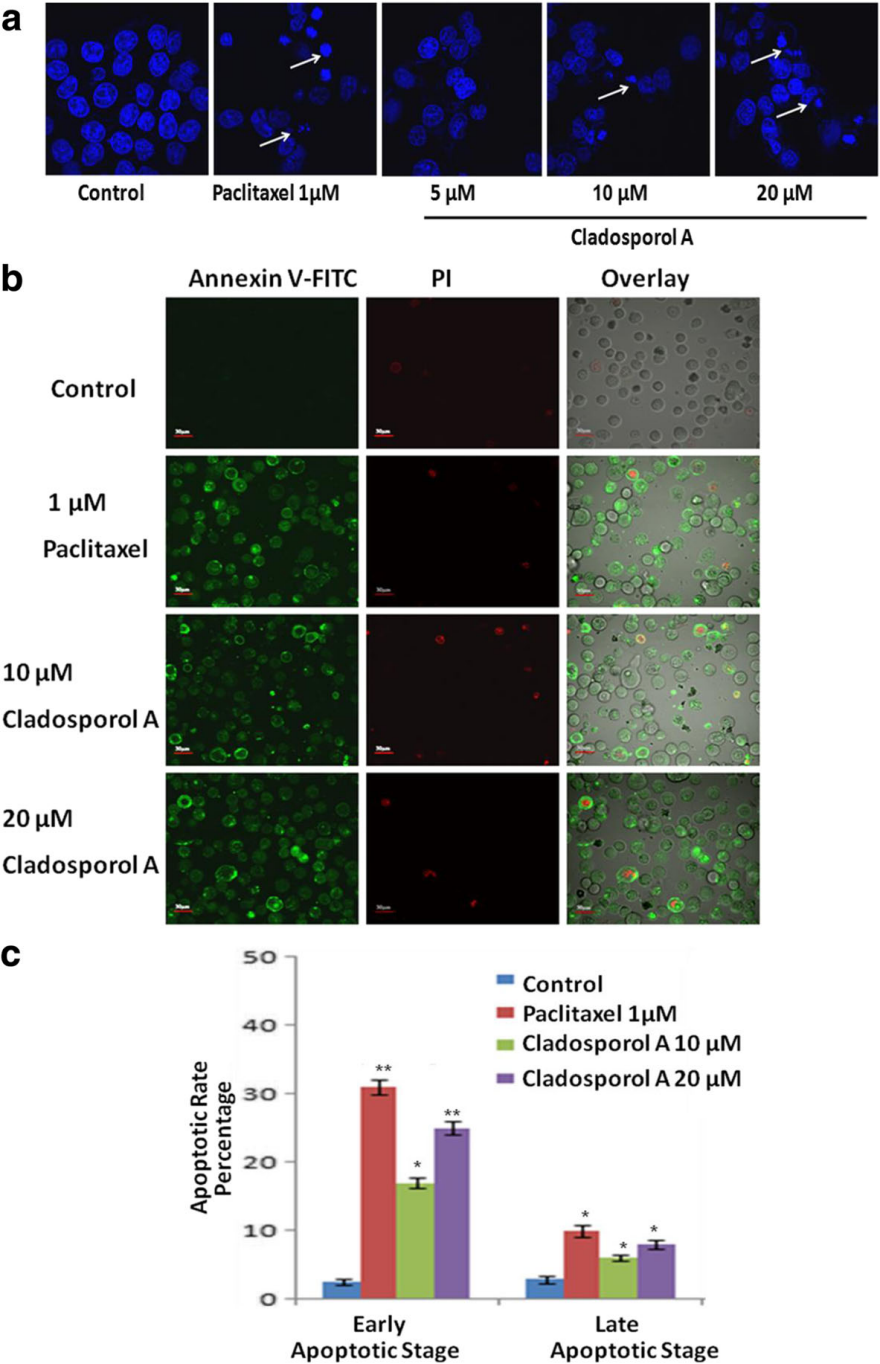
**a** Nuclear morphology analysis of human breast cancer (MCF-7) cells (2 × 10^5^/ml/well) using DAPI after treatment with different concentrations of Cladosporol A (5, 10, 20 μM) for 24 h and examined using fluorescence microscopy (40X). Paclitaxel (1 μM) was used as positive control. With increase in concentration of Cladosporol A there is significant increase in nuclear condensation and formation of apoptotic bodies (**b**). The effect of Cladosporol A on the exposure of phosphatidylserine (PS) in human breast cancer (MCF-7) cells after 24 h treatment was analysed. Phosphatidylserine exposure was assessed by the annexin-V/ propidium iodide assay, as described in methodology and analyzed by confocal microscopy (**c**). The percentage of cells in early and late stages of apoptosis obtained by analysis of the cell images (mean ± SD, 3 experiments), p*< 0.05 and **p < 0.01

Apoptosis induction in human breast cancer (MCF-7) cells via Cladosporol A treatment was determined microscopically by analysis of DAPI stained cells. Cladosporol A significantly induced condensation of chromatin and its fragmentation within the nucleus in concentration dependent manner in MCF-7 cells after treatment of 24 h. Promising chromatin condensation was also observed in paclitaxel treated cells (Fig. 6a). Further, a specific marker which detects the phosphatidylserine externalization during apoptosis, a phosphatidylserine-binding protein (annexin V) was utilized to assess the extent of apoptosis of human breast cancer (MCF-7) cells after Cladosporol A treatment. The results of Annexin V-FITC and PI dual staining suggested that both the two dyes did not stained the normal cells, whereas Annexin V-FITC stained only the early apoptotic cells and both Annexin V-FITC and PI stained the late apoptotic cells. As it is evident from Fig. 6b, maximum of the Cladosporol A and paclitaxel treated cells were Annexin V-FITC (green) stained only indicating their early apoptotic stage. Additionally, some percentage of the cells, treated with Cladosporol A and paclitaxel, were stained by both dyes indicating that these cells were in late apoptotic stage. In Fig. 6c, at lower concentration (10 μM) Cladosporol A induces 16% early stage apoptosis and 6% of late stage apoptosis. At 20 μM concentration Cladosporol A induces 25% early stage apoptosis and 9% late apoptotic stage.

### Cladosporol A induces ROS-mediated cell death

**Fig. 7:**
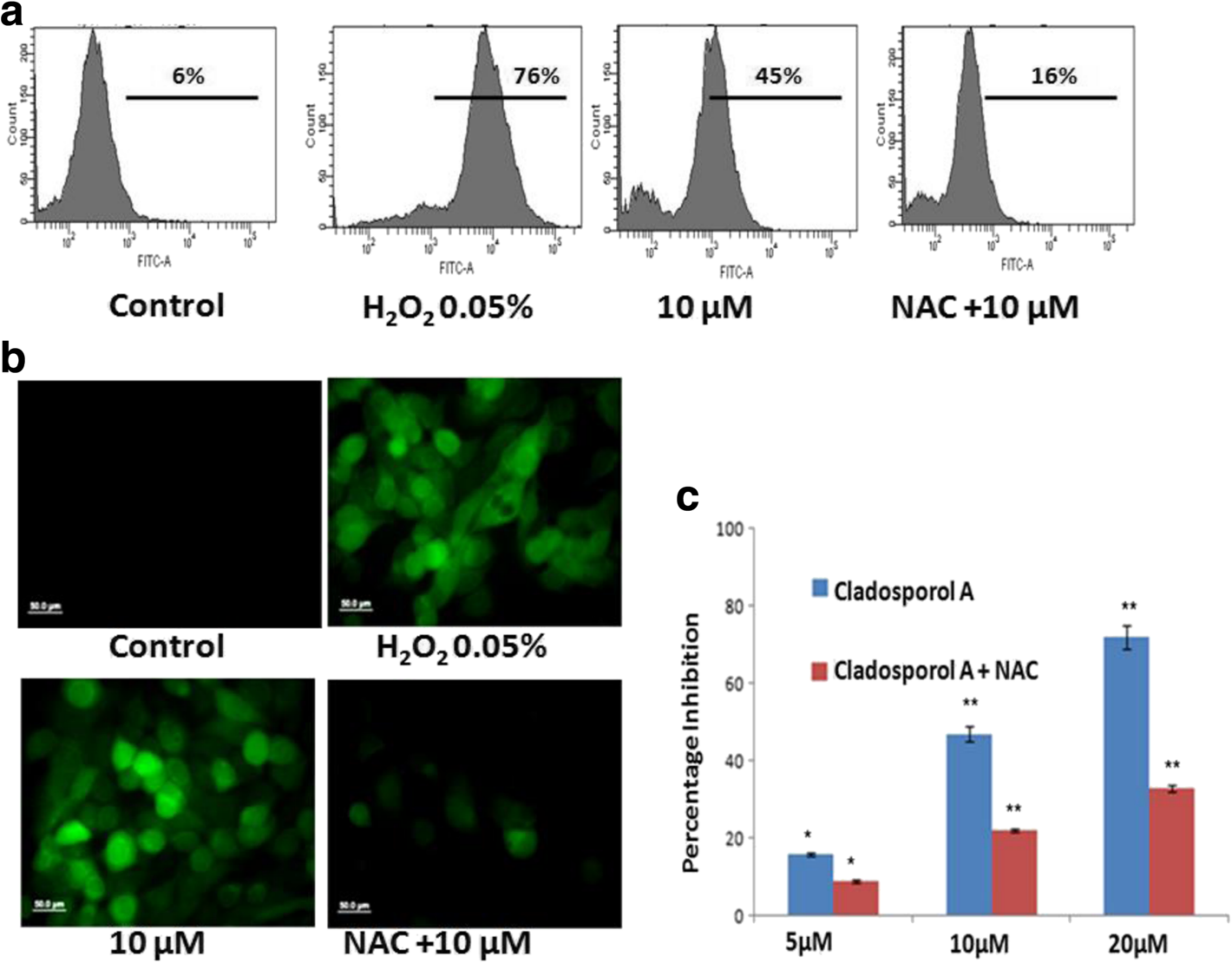
**a** Intracellular ROS level was measured by flow cytometry analysis using DCFH- DA after 24 h. Human breast cancer (MCF-7) cells were treated with indicated concentrations of Cladosporol A and 0.05% H_2_O_2_ and incubated with 5 μM DCFH-DA. **b** Detection of ROS by fluorescence microscope. After incubation with DCFH-DA and 0.05% H_2_O_2_, MCF-7 cells (2 × 10^5^/ml/well) were washed and examined by fluorescence microscope (40X). **c** MCF-7 cell growth inhibition in the presence of ROS scavenger (NAC) was also determined. The cells were treated with indicated concentrations of Cladosporol A in the presence or absence of NAC (150 μM)

Subsequent studies were conducted to assess levels of reactive oxygen species (ROS) following treatment with Cladosporol A measured by the fluorescent indicator ′,7′-dichlorofluorescein diacetate. After loading human breast cancer (MCF-7) cells with 2′,7′-dichlorofluorescein diacetate a ROS-sensitive fluorescent probe, intracellular ROS levels were measured by flow cytometry and fluorescence microscope (Fig. 7a & b). ROS generation was induced in a significant manner by Cladosporol A treatment, as indicated by the increase in fluorescence intensity. In comparison with untreated cells, nearly 45% ROS was generated in the cells treated with 20 μM Cladosporol A after 24 h treatment. Furthermore, to determine the involvement of ROS generation in cell death induced by Cladosporol A, ROS accumulation as well as cell viability was studied in the presence or absence of NAC (N-acetyl-cysteine) (ROS scavenger) was performed. In the presence of NAC, Cladosporol A treatment was unable to elevate ROS levels (Fig. 7a & b) and failed to induce cell death significantly as well (Fig. 7c), indicating that cell death was ROS-mediated.

### Cladosporol A induces loss in mitochondrial membrane potential (ΔΨm) and causes cytochrome c release in human breast cancer (MCF-7) cells

**Fig. 8:**
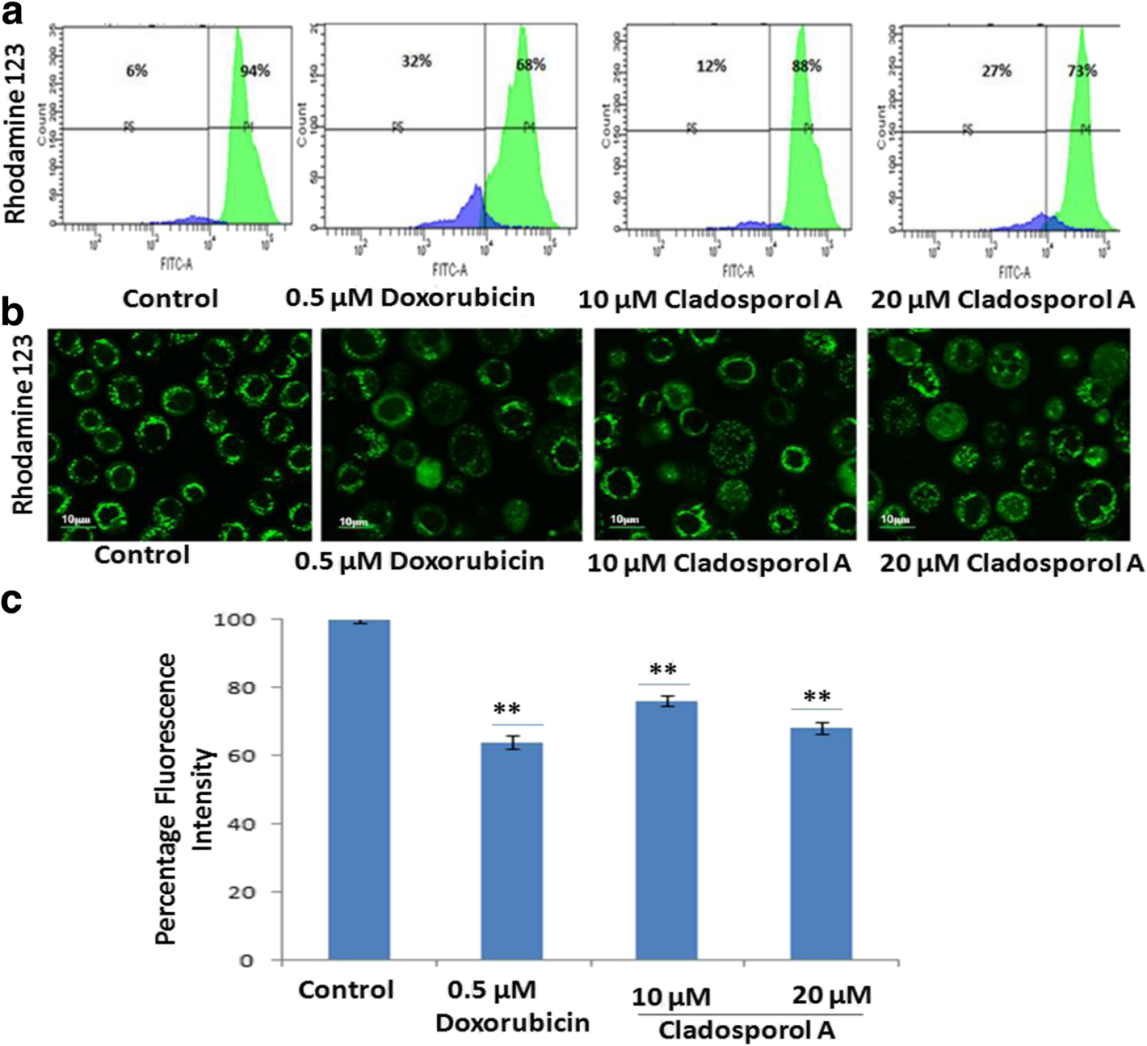
Cladosporol A induces loss of mitochondrial transmembrane potential. Human breast cancer (MCF-7) cells were incubated with different concentrations of Cladosporol A for 24 h. Thereafter, cells were stained with Rh-123 (1 μM) for 20 min and analyzed by (**a**) Flow cytometer (**b**)**.** Confocal microscopy (**c**)). Histogram showing the effect of Cladosporol A on Δψm measured with laser scanning confocal microscope by staining with Rhodamine 123. Data are presented as mean ± S.D., statistical analysis was done with **p < 0.01

**Fig. 9:**
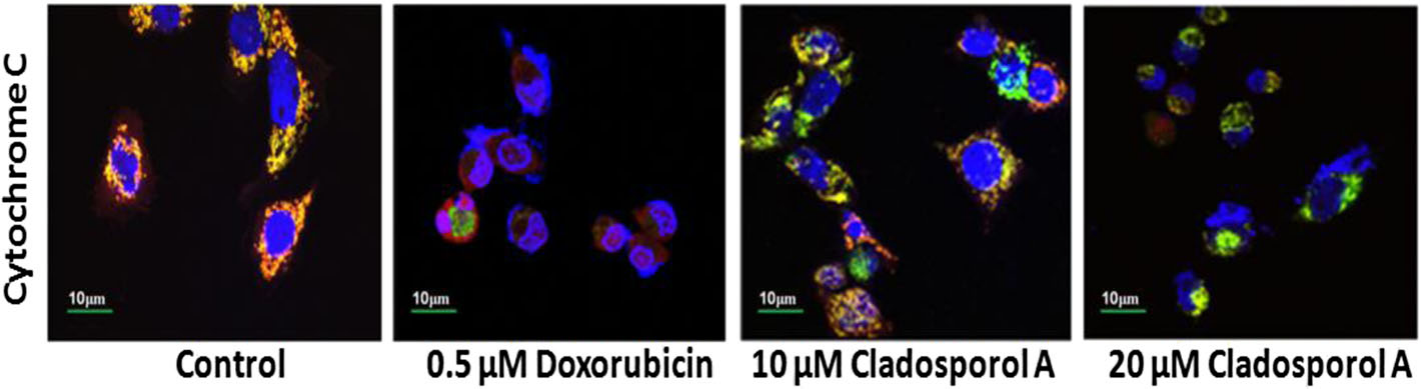
Colocalization of cytochrome c and mitochondria was determined by confocal microscopy. Human breast cancer (MCF-7) cells were immunostained for cytochrome c release (green) and the mitochondria of the cells were stained with MitoTracker (red). MCF-7 cells were treated with different concentrations of Cladosporol A and doxorubicin (500 nM) for 24 h and stained with anti-cytochrome c antibody and Alexa Fluor 488-labeled secondary antibody

Flow cytometry and laser scanning confocal microscope (LSCM) were used to measure of mitochondrial membrane potential (ΔΨm) loss (Fig. 8 a & b) with Rh123 staining. Human breast cancer (MCF-7) cells after Cladosporol A treatment for 24 h induced a significant mitochondrial membrane potential loss in human breast cancer (MCF-7) cells in a concentration dependent manner. Doxorubicin (positive control) treated human breast cancer (MCF-7) cells also showed a significant reduction in mitochondrial membrane potential. These data suggested that Cladosporol A induces apoptosis via MMP (ΔΨm) loss. In Fig. 8 C Flow cytometric analysis showed that Cladosporol A at 20 μM concentration induces 27% of MMP loss while at 10 μM concentration causes 12% of the MMP loss. Colocalization between mitochondria and cytochrome c was studied by laser scanning confocal microscopy. MitoTracker red dye was used to label functional mitochondria. Localization of cytochrome c was ascertained by immunofluorescence using a specific antibody of cytochrome c. In control cells, cytochrome c was colocalized with mitochondria (Fig. 9). In contrast, Cladosporol A and doxorubicin treatments induced a significant reduction in mitochondria and release of cytochrome c to the cytosol respectively.

### Cladosporol A treatment induces upregulation of Bax and downregulation of Bcl-2 expression in human breast cancer (MCF-7) cells

**Fig. 10:**
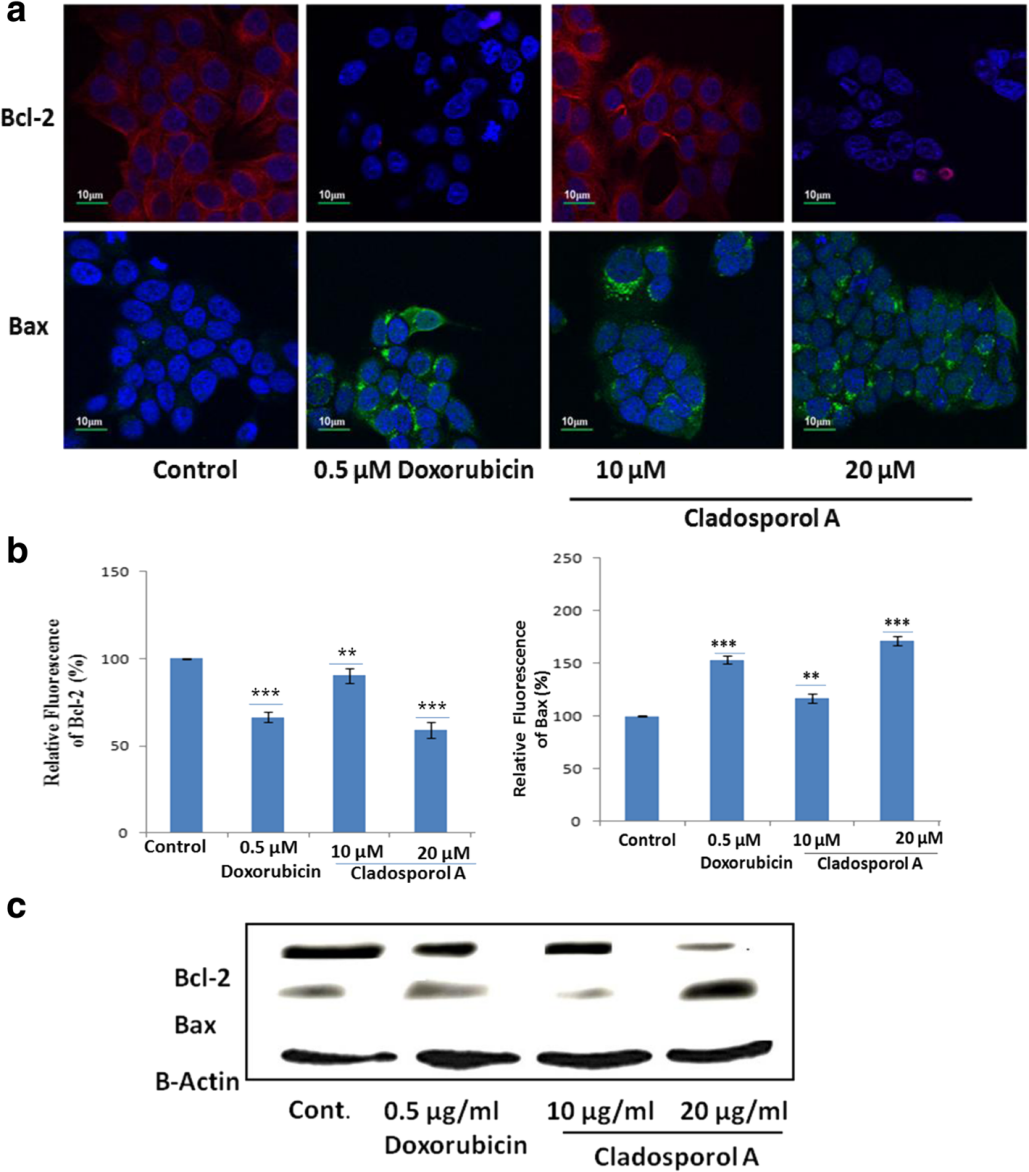
Effects of Cladosporol A and doxorubicin treatment on the expression of apoptosis related proteins. (**a**) Representative images of immunofluorescence analysis of effects of the expression of; antiapoptotic Bcl-2 and pro-apoptotic Bax by confocal microscopy (using 40× oil immersion lens) in human breast cancer (MCF-7) cells treated with Cladosporol A for 24 h (**b**). Statistical analysis of the expression of Bcl-2 and Bax. The relative fluorescence intensity was compared to the control group. Data are mean ± S.D. of three similar experiments; statistical analysis was done with **p < 0.01 and ***p < 0.001. **c** Western blot indicating the increase in bax and decrease bcl-2 protein expression after Cladosporol A treatment in concentration dependent manner

Pro-apoptotic protein Bax and anti-apoptotic Bcl-2 plays a very significant role in mitochondrial mediated apoptotic pathway. The expression levels of Bax and Bcl-2 in human breast cancer (MCF-7) cells after Cladosporol A treatement for 24 h were evaluated, using immunofluorescence microscopic studies and western blot analysis. Results of immunofluorescence microscopy and western blot analysis suggested that the fluorescence intensity of Bcl-2 reduced whereas in case of Bax increased after Cladosporol A treatment in concentration dependent manner (Fig. 10 a and c). Thus, apoptosis induction via Cladosporol A, was accompanied by upregulation of Bax and downregulation of Bcl-2 in a concentration dependent manner.

### Cladosporol A treatment also induces autophagic cell death in human breast cancer (MCF-7) cells

**Fig. 11:**
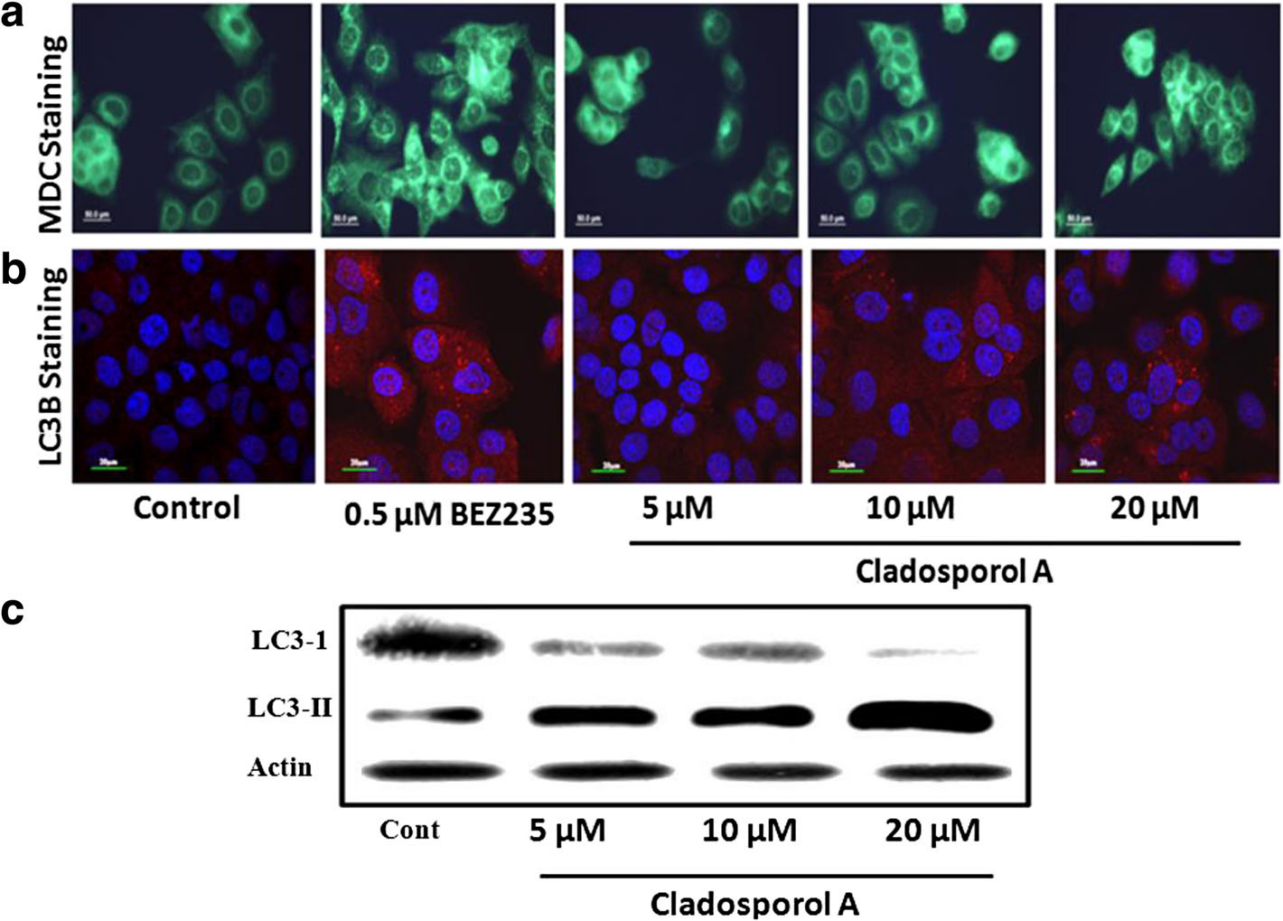
Cladosporol A induces autophagy in human breast cancer (MCF-7 cells) (**a**). The autophagic vacuoles were observed under fluorescence microscope (40×) with MDC staining. The treatment of Cladosporol A and BEZ235 (positive control group) induced concentration-dependent formation of autophagic vacuoles in MCF-7 cells after 24 h. **b** Detection of autophagy with LC3b antibody using confocal microscopy. Immunocytochemical staining was conducted using anti-LC3b antibody and Alexa Flour-555-labelled secondary antibody. Nuclei were stained with DAPI. **c** Detection of autophagy by western blot analysis after Cladosporol A treatment in concentration dependent manner

Changes in the fluorescence intensity of MDC (monodansylcadaverine staining), a specific marker for autophagic vacuoles was used to detect the autophagic activity of Cladosporol A (Fig. 11a). Compared to the untreated control cells, in Cladosporol A treated human breast (MCF-7) cancer cells, the number of autophagic vacuoles stained by MDC were much higher. Furthermore, to mechanistically confirm the Cladosporol A induced autophagy induction, a set of autophagy-related factors including LC3-I and LC3-II in the human breast (MCF-7) cancer cells were investigated by immunofluorecence microscopy and western blot analysis after treatment with Cladosporol A at various concentrations for 24 h (Fig. 11 b and c). Cladosporol A treatment significantly induced conversion of LC3-I to LC3-II.

## Discussion

Natural products represent an unsurpassed source of bioactive compounds and constitute a relevant resource for the pharmaceutical industry. Endophytes represent a potential source for isolation of variety of lead compounds for the development of drugs for various malignancies. Breast cancer is the frequently diagnosed malignancy leading to cancer related deaths worldwide. Various gene mutations taking place in luminal or basal progenitor cell population giving rise to phenotypically heterogeneous cell population is responsible for breast cancer [16]. There is a need to develop novel strategies for achieving additional means to control development and progression of breast cancer. In the search for novel anticancer agents against breast cancer with effective chemotherapeutic spectrum various research groups have been motivated to isolate and characterize natural compounds from fungal endophytes that will increase the number of these therapeutic tools to inhibit breast cancer cells proliferation [17]. In the present study, Cladosporol A, a potent, natural compound was isolated and purified from endophytic fungus *Cladosporium cladosporioides* isolated from *Datura innoxia* plant. *Cladosporium cladosporioides* is main producer of antifungal metabolites i.e. cladosporin, 5 hydroxyasperentin and isocladosporin. These molecules (metabolites) have shown promising activity in the treatment and control of various plant-infected diseases [18]. We determined the antiproliferative activity of the Cladosporol A, a potent, natural compound isolated against NCI60 human cancer cell lines. It has shown least IC_50_ value of 8.7 μM against human breast (MCF-7) cancer cells (Table 1). We next ascertained the effect of Cladosporol A treatment on proliferation, growth and clonogenic ability of human breast (MCF-7) cancer cells. Cladosporol A treatment produced a concentration-dependent inhibitory effect on the ability of human breast (MCF-7) cancer cells to reproduce and form large colonies (Fig. 4 a & b). We therefore used human breast (MCF-7) cancer cell line as a model cell line for further mechanistic studies. We examined the mechanism of changes in cell cycle distribution caused by Cladosporol A in MCF-7 cells. It causes G0/G1 phase arrest and also induces accumulation of some cells in the G2/M phase (Fig. 5a). Microtubules play an important role in different phases of the cell cycle. Their differential dynamic behaviour has qualified them as targets for treating several diseases including cancer. Several antimicrotubule agents have been evaluated for their promising uses in cancer chemotherapy e.g. paclitaxel, vinblastine, griseofulvin etc. These agents have shown to induce apoptosis via targeting microtubule assembly dynamics [19]. Immunofluorescence confocal microscopic studies on MCF-7 cells after Cladosporol A treatment revealed that it induces disruption of microtubules by inhibiting microtubule polymerization in concentration-dependent manner (Fig.5b)*.* Further, we systematically analyzed the involvement of p21 in Cladosporol A mediated cell death. Our immunofluorescence studies as well as western blot analysis revealed increase in p21 protein expression in human breast (MCF-7) cancer cells after Cladosporol A treatment in a concentration dependent manner (Fig. 5c and d). Further the induction of apoptosis in human breast cancer (MCF-7) cells after Cladosporol A treatment was determined microscopically by analysis of DAPI stained cells. Cladosporol A treatment induced promising condensation of chromatin and its fragmentation within the nucleus in concentration dependent manner after 24 h treatment (Fig. 6a). Next, Annexin V-FITC/PI dual staining was performed to further determine the molecular mechanism of Cladosporol A induced cell death (apoptosis) in human breast (MCF-7) cancer cells by laser scanning confocal microscopy. The data indicated that following Cladosporol A treatment annexin V-FITC positive cells were seen in a significant amount whereas untreated cells were negative for annexin V-FITC and PI staining (Fig. 6b). ROS plays a vital role in various cellular biological activities including the processes of tumor metastasis and progression. In comparison to normal cells, cancer cells usually possess elevated ROS levels as well as antioxidant activities in an uncontrolled manner [20, 21]. Earlier it has been elucidated that targeting ROS is a critically significant cancer therapeutic strategy. This has promisingly contributed to the potent anticancer effects of paclitaxel thereby improving its clinical use. Thus, anticancer agents which induce the production of ROS are useful for eliminating the bulk of cancer cells [22]. We determined ROS generation induced by Cladosporol A in human breast cancer (MCF-7) cells. Cladosporol A induced prominent ROS generation in concentration dependent manner (Fig. 7a&b). To determine the role of ROS in inducing apoptosis, we tested the effect of NAC on Cladosporol A treated human breast cancer (MCF-7) cells and found that ROS generation was almost completely inhibited by NAC pretreatment of cells treated with Cladosporol A for 24 h (Fig. 7c). Mitochondria play a pivotal role as energetic centres and contain various pro-apoptotic molecules that further activate cytosolic proteins to initiate apoptosis. The mitochondrial dysfunction and changes in the ΔΨm are considered an early event in apoptosis [23, 24]. In the cell major source of oxidative stress is mitochondria-mediated ROS generation [4, 25]. Flow cytometery and Laser scanning confocal microscopy (LCMS) results suggested that human breast (MCF-7) cancer cells treated with different concentrations of Cladosporol A (Fig. 8 a, b,c) considerable caused significant mitochondrial membrane potential loss. To further mechanistically investigate the apoptotic pathway, cytochrome c release induced by Cladosporol A treatment was studied. The results of our immunofluorescence data revealed, cytochrome c to be highly colocalized with mitochondria, in untreated control cells. In contrast, treatment of human breast (MCF-7) cancer cells with Cladosporol A and positive control (doxorubicin) caused diffused cytoplasmic distribution of cytochrome c from the transition pores of the mitochondria into the cytosol (Fig. 9). The best characterized protein family that play an important role in apoptotic cell death regulation are Bcl-2 family proteins. The ratio of pro- to anti-apoptotic proteins, of the Bcl-2 family proteins critically mediate mitochondrial cell death pathway [26]. Disruption of ΔΨm and cytochrome c excretion into the cytosol is promoted by Bax, the pro-apoptotic protein on the other hand mitochondrial integrity is preserved by Bcl-2, an anti-apoptotic thereby preventing the apoptosis [27]. Bax and Bcl-2 balance is thus, very critical in apoptotic pathway mediated by mitochondrial dysfunction [28]. Our results reflected the involvement of Bcl-2 family proteins in Cladosporol A mediated cell death. Treatment of human breast cancer (MCF-7) cells with Cladosporol A decreased ΔΨm, upregulated Bax expression and downregulated Bcl-2 levels in a concentration-dependent manner, thereby increasing the ratio of Bax/Bcl-2 protein levels (Fig. 10). Thus the study clearly revealed the involvement of mitochondrial mediated pathway in Cladosporol A induced cell apoptosis. Several anticancer agents are known to induce tumor growth inhibition thereby causing mitophagic cell death [29]. Furthermore, another target for cancer treatment is autophagy. It has provided new opportunity for discovery and development of novel cancer therapeutics. To elucidate the role of Cladosporol A in mediating autophagic cell death autophagolysosomes formation induced by it was examined by staining human breast cancer (MCF-7) cells by MDC (monodansylcadaverine), a tracer for autophagic vacuoles [30]. Cladosporol A treatment significantly increased MDC fluorescence in a concentration-dependent manner (Fig. 11a). Furthermore, the microtubule associated protein light chain 3 (LC3) is the another signature marker of autophagosomes. A key step in autophagy is the cleavage of the 18 kDa full length LC3, known as LC3-I, to a 16 kDa form, known as LC3-II, resulting in its recruitment to double-layered membrane of autophagosomes [31]. Our immunofluorescence confocal microscopic studies revealed that human breast (MCF-7) cancer cells treated with Cladosporol A significantly showed concentration-dependent increase in conversion of LC3-I to LC3-II (Fig. 11b and c).

## Conclusions

Present data indicated that Cladosporol A isolated from endophytic *Cladosporium cladosporioides* from *Datura innoxia* mechanistically showed marked growth inhibition against panel of NCI60 human cancer cell lines especially against human breast (MCF-7) cancer cells. It triggers ROS-mediated mitophagic cell death inducing loss in MMP, releasing cytochrome c, in turn up regulating the expression of Bax and down regulating the level of Bcl-2 proteins sensitizing apoptosis via autophagic flux due to increased conversion of LC3-I to LC3-II. It also causes microtubule depolymerisation and increase in p21 protein expression. Our study provides a molecular basis for its development as novel anticancer lead for human breast cancer.

## Methods

### Chemicals and antibodies

PDA (Potato Dextrose Agar), lactophenol-cotton-blue, 1% sodium hypochlorite, Glycerol 15% was obtained from Difco. From Qiagen Purification kit for DNA was obtained, DNA MiniPrep™ kit (Sigma), HiPurA™ PCR product purification kit (HiMedia Laboratories), Penicillin G, dimethyl sulfoxide (DMSO), streptomycin, trypsin-EDTA, 3-(4,5-dimethylthiazole-2-yl)-2,5-diphenyltetrazolium bromide (MTT), Propidium iodide (PI), 2′,7′-dichlorofluorescein diacetate, DNase-free RNase, paraformaldehyde, annexin V-FITC Kit, crystal violet, N-acetyl cysteine, doxorubicin and paclitaxel were purchased from Sigma Chemicals Co. (St. Louis, MO). DAPI mounting medium was purchased from Cell signaling technology (CST). Fetal bovine serum (10%) was purchased from Gibco. Antibodies were purchased from different commercial sources: Anti cytochrome c, anti p21, anti bax and anti bcl-2 were procured from Santa Cruz biotechnology incorporation and anti α-tubulin was procured from Sigma Chemical, St. Louis, MO. Secondary antibodies were obtained from CST. Other reagents were of analytical grade and were purchased from local sources.

### Cell lines, cell culture, growth conditions and treatment

Colon (HCT-116) cancer cells, ovarian (OVCAR-3) cancer cells, lung (A549) cancer cells, breast (MCF-7) cancer cells and prostate (PC3) cancer cells, all were procured from NCI (National Cancer Institute) and were cultured in RPMI 1640/ McCoy’s supplementation of medium was done with penicillin (100 units/ml), streptomycin (100 μg/ml), 10% FBS (fetal bovine serum), sodium pyruvate (550 mg/ml), L-glutamine (0.3 mg/ml) and NaHCO_3_ (2 mg/ml). Cells were cultured at 37^o^ C in CO_2_ incubator (Heraeus, GmbH, Germany) with 95% humidity and 5.0% CO_2_. In DMSO Cladosporol A was dissolved and cells were treated with it, while only vehicle was added to untreated control cultures. The concentration of DMSO added to the cultures was maintained at <1%.

### Isolation of endophytic fungi

Root sample of *Datura innoxia* (Family: Solanaceae) was collected in January 2013, from healthy and symptomless mature plant, located in Jammu (32.72°N, 74.77°E), India. Sample was taken in sterilized polythene bag kept in icebox and processed within 24 h of collection. Sample was washed with the running tap water followed by thrice washing with autoclaved distilled water and air dried. Isolation of endophytic fungi was done by the procedure as described in our previous work [4]. The mycelium was picked and transferred to potato dextrose agar media (PDA), incubated at 28 °C and pure culture was obtained. The pure culture of the endophytic fungus DIE-10 was deposited to the Col Sir R. N. Chopra, Microbial Resource Center Jammu (MRCJ), India under accession number MRCJ-314.

### Identification, characterization and phylogenetic analysis of endophytic fungus MRCJ-314

ZR Fungal/Bacterial DNA MiniPrep™ kit was used to obtain the pure genomic DNA. Spectrophotometrically (NanoDrop 2000) analyzed DNA was used for the PCR amplification of ITS1–5.8S–ITS2 regions. PCR was performed by universal primers ITS5: 5′-GGAAGTAAAAGTCGTAACAAGG-3′ and ITS4: 5′-TCCTCCGCTTATTGATATGC-3′ [32]. The thermal cycling program used was as per our previous study [4]. The sequences obtained were used as query sequence for similarity search by using BLAST algorithm against the database maintained at NCBI. The ITS region sequence of (DIE-10) MRCJ-314 isolate was aligned with the most similar reference sequences of the taxa by using the clustal W module of MEGA6 software [33]. A phylogenetic tree was constructed subsequently analyzed for evolutionary distances by the neighbor joining method. The robustness of clades was determined by analysis of bootstrap with 1000 replications. The contiguous rDNA sequences of the representative isolate have been deposited in GenBank under the accession number KX553964.

### Fermentation and isolation of Cladosporol A

The fungus was fermented in potato dextrose broth (PDB) medium for 7 days, pH 5.5 at 28 °C in dark with constant shaking in six 1 L flasks containing 300 ml broth. The fermentation broth was then extracted with dichloromethane following the National Cancer Institute’s protocol [34]. The resulting extract was then concentrated in vacuum and subjected to column chromatography on silica gel using hexane-ethyl acetate with increasing polarity leading to the isolation of cladosporol A (Fig 2). The ^1^H and ^13^C NMR data of cladosporol A was found to be in identical range to that of reported in literature [35]. The purity of cladosporol A was established by HPLC: tR = 34.4 min (>99% purity) (please see Additional file 1: Figure S1 for spectral graphs).

### Analytical data of Cladosporol A

White Solid, ^1^H NMR (400 MHz, CDCl_3_) δ 12.59 (d, *J* = 6.7 Hz, 1H), 8.62 (s, 1H), 7.33–7.20 (m, 2H), 7.01 (t, *J* = 15.8 Hz, 1H), 6.95 (d, *J* = 8.6 Hz, 1H), 6.80 (d, *J* = 8.3 Hz, 1H), 6.23 (d, *J* = 7.6 Hz, 1H), 5.43 (d, *J* = 8.7 Hz, 1H), 4.88 (dd, *J* = 8.2, 4.8 Hz, 1H), 4.10–4.04 (m, 1H), 3.87 (d, *J* = 4.5 Hz, 1H), 3.53 (t, *J* = 14.0 Hz, 1H), 2.76 (t, *J* = 6.6 Hz, 2H), 2.51 (dt, *J* = 18.7, 5.5 Hz, 1H), 2.21 (dt, *J* = 22.4, 8.2 Hz, 1H); ^13^C NMR (125 MHz, CDCl_3_) δ 205.3, 194.9, 162.7, 137.3, 136.4, 132.2, 122.5, 120.0, 115.8, 67.5, 56.1, 55.2, 40.1, 36.8, 31.0. HRMS (ESI^+^) calculated for C_20_H_16_O_6_ [M ± H]^+^: 353.1020, found: 353.1015.

### Cell viability (MTT) assay

MTT dye was used for performing cell proliferation assay. Various human cancer cell lines at a density of 8 × 10^4^ cells/well were seeded in 96-well culture plates. Cells were incubated with various concentrations of Cladosporol A after 24 h and untreated cells served as control. After 48 h of incubation of the test material, MTT dye, 3[4,5-dimethylthiazol- 2-yl]-2,5-diphenyl-tetrazolium bromide, was then added before 4 h of the experiment termination (2.5 mg/mL). The resulting MTT formazan crystals obtained were dissolved in DMSO (150 ml) and at 570 nm (reference wavelength 620 nm), OD was measured. Further, by comparing the absorbance of treated and untreated cells, percentage viability (cell growth) was calculated [36].

### DNA content and cell cycle phase distribution

Human breast (MCF-7) cancer cell line at a seeding density of 2 × 10^5^ cells/well, were seeded in six-well plates and treated with 5, 10 and 20 μM concentrations of Cladosporol A and 1000 nM concentration of paclitaxel. Treated cells were harvested after 24 h, PBS washed and fixed overnight at −20 °C in 70% cold ethanol. Later on, cells were again given PBS washing and further treated for RNase digestion (0.1 mg/ml) at 37 °C for 90 min. PI (50 μg/ml) was used to stain the cells and with the help of flow-cytometry (BD FACS Aria) further analysis of the relative DNA content was done [37].

### Nuclear morphology analysis

The presence of apoptotic cells were determined by staining MCF-7 (human breast cancer cells) with DAPI. MCF-7 cells were seeded in 60 mm culture dishes at a seeding density of 2 × 10^5^cells/well and incubated for 24 h. After that cells were treated with various concentrations of Cladosporol A (5, 10, 20 μM) and again incubated for 24 h. The media was decanted, collected and PBS wash was given to the cells. Trypsinization was carried out to detach the adherent cells. In the analysis collectively, floating and poorly attached cells were included. Cells were centrifuged and air-dried smears of MCF-7 cells were prepared. Later on in methanol at −20 °C, air-dried smears of MCF-7 cells were fixed for 20 min and DAPI (1 μg/ml in PBS) stained for 20 min at room temperature in the dark. Glycerol-PBS (1:1) was used to mount the slides and prepared slides were observed via inverted fluorescence microscope (Olympus, 1X81) [4, 38].

### Apoptosis detection by Annexin V-FITC and PI dual staining

To investigate the apoptosis-inducing effect of Cladosporol A, we analyzed the percentage of early and late apoptotic cells by Annexin V-FITC and propidium iodide (PI) dual staining. Human breast (MCF-7) cancer cells at a seeding density of 2 × 10^5^ cells/well, were seeded in six-well plates. After treatment with different concentrations of Cladosporol A (10 and 20 μM) and paclitaxel (1 μM) for 24 h, harvested cells were PBS washed two times and suspended in binding buffer. After that, Annexin V/FITC and PI was used to stain the cells in dark for 15 min and cells were further observed by LSCM (laser scanning confocal microscope) [4, 39].

### Determination of intracellular ROS

To identify the role of ROS in mediating Cladosporol A anti-cancer effects, intracellular ROS levels were monitored using 2′,7′-dichlorofluorescein diacetate (DCFH-DA). Human breast cancer (MCF-7) cells at a seeding density of 2X10^5^ cells/well were seeded in a six well plate and incubated overnight. After that, cells were treated with Cladosporol A for 24 h and H_2_O_2_ was used as a positive control. The cells were then treated with DCFH-DA (5 μM) at 37 °C for 30 min. Further, flow cytometry and fluorescence microscopy was used to measure the fluorescence intensity of the treated cells [4, 25].

### Loss of mitochondrial membrane potential (MMP)

Alterations in MMP (Δψm) were determined, by using the fluorescent dye rhodamine 123 (Rh-123) staining method and were studied using confocal microscopy. Human breast cancer (MCF-7) cells at a seeding density of 2 × 10^5^ cells/well were seeded in 6-well plates and treated with different concentrations of Cladosporol A (10 and 20 μM) and doxorubicin (0.5 μM /mL) for 24 h. Later on, trypsinization was carried to detach the cells and resulting cell pellets were given two times PBS washing. 2 ml fresh medium containing Rh123 (Rhodamine 123) (1.0 μM) was then added to the cell pellets and were incubated at 37 °C for 20 min with gentle shaking. The cells were centrifuged and resulting cell pellets were washed again with PBS two times, then observed using a laser scanning confocal microscope (Olympus Fluoview FV1000) [4, 40].

### Autophagy analysis by monodansylcadaverine staining

Development of autophagic vacuoles determines the level of autophagy. Monodansylcadaverine (MDC) has been regarded as a specific marker for the detection of autophagic vacuoles. By treating the cells with MDC (0.05 mM) in PBS at 37 °C for 1 h, autophagic vacuoles were labelled. After completion of incubation, Cladosporol A treated cells were washed thrice with PBS and immediately observed by fluorescence microscope using 40× lens [41].

### Detection of p21, α-tubulin, cytochrome c, Bcl-2, Bax and LC3b by immunofluorescence confocal microscopy

Human breast cancer (MCF-7) (2 × 10^5^ cells/well) cells, after treatment with different concentrations of Cladosporol A (10 and 20 μM) and doxorubicin (0.5 μM /ml) were processed for immunofluorescence microscopic studies. Briefly the cells were grown on cover slips, 4% paraformaldehyde was used to fix the cells for 10 min at room temperature and then cells were permeabilized using 0.5% Triton-X in PBS for 5 min. 10% goat serum was used to block the cells for 20 min at room temperature. The expression of proteins such as p21, α-tubulin, cytochrome c, Bcl-2, Bax and LC3b was observed by treating the cells for 1 h at room temperature with their specific primary antibodies. Cells were then incubated for 1 h at room temperature with respective conjugated secondary antibodies, alexa fluor 488 and 555. Further, the cells were given PBS washing thrice and were further stained with DAPI (1 μg/ml in PBS). Then over glass slides, cover slips were mounted and cells were observed and imaged via laser scanning confocal microscope (LSCM) [4, 42, 43].

### In vitro clonogenic assay

To test the effect of Cladosporol A treatment on the colony formation ability of MCF-7 cells, clonogenic assay was performed. MCF-7 cells were seeded at 1 × 10^3^ cells/ml/well in 6-well plates. The culture medium was changed after 24 h, and fresh medium was added, and Cladosporol A (5, 10, 20 μM) treatment was given to cells for 7 days in a 37 °C incubator in 5% CO_2_. Thereafter, 4% paraformaldehyde was used to fix the obtained colonies which were further stained with crystal violet solution (0.5%). Thereafter, from the plates colonies were observed, counted and photographed [4, 11, 44].

### Western blot analysis

Cladosporol A treated MCF-7 cells and untreated control cells were centrifuged at 4 °C at 400 g. The resulting cell pellets were given PBS washing and whole-cell lysate was prepared. The cell pellets were further lysed with RIPA buffer. Thereafter, for SDS-PAGE, equal amount of protein (60 μg) was loaded into each well. Further, primary antibodies; p21, Bcl-2 and Bax, LC3b were used to incubate the resulting blots and chemiluminiscence was captured on hyper film after incubating the blots in ECL plus solution [37].

### Statistical analysis

Three independent experiment results were expressed as mean ± SD. Statistical analysis was performed using an unpaired t test, *p < 0.05, **p < 0.01, and ***p < 0.001.

## Additional file

Additional file 1:
**Figure S1.** ^1^H–NMR of Cladosporol A. **Figure S2.** ^13^C–NMR of Cladosporol A. **Figure S3.** HPLC spectra of Cladosporol A. (DOC 374 kb)

## Abbreviations

DAPI: 4,6-diamidino-2-phenylindole
DMSO: Dimethyl sulfoxide
FBS: Fetal bovine serum
MTT: 3-(4,5-dimethylthiazole-2- yl)-2,5-diphenyltetrazolium bromide
NAC: N-acetyl-cysteine
PDA: Potato dextrose agar
PI: Propidium iodide
ROS: Reactive oxygen species
