# Erratum to: Cladosporol a triggers apoptosis sensitivity by ROS-mediated autophagic flux in human breast cancer cells

**DOI:** 10.1186/s12860-017-0143-y

**Published:** 2017-08-04

**Authors:** Mytre Koul, Ashok Kumar, Ramesh Deshidi, Vishal Sharma, Rachna D. Singh, Jasvinder Singh, Parduman Raj Sharma, Bhahwal Ali Shah, Sundeep Jaglan, Shashank Singh

**Affiliations:** 1 Cancer Pharmacology Division, CSIR-Indian Institute of Integrative Medicine, Jammu, India; 2 Natural Product Chemistry, CSIR-Indian Institute of Integrative Medicine, Jammu, India; 3 Microbial Biotechnology Division, CSIR-Indian Institute of Integrative Medicine, Jammu, India; 4 Academy of Scientific &Innovative Research (AcSIR), CSIR, New Delhi, India; 5 Department of Conservative Dentistry & Endodontics, Indira Gandhi Govt. Dental College and Hospital, Jammu, India

## Erratum

The original article [1] was corrected. Corresponding author: Bhahwal Ali Shah’s name was originally spelt incorrectly. It has been changed from Bhawal to Bhahwal.
