# Supplementary material for: Cladosporol A triggers apoptosis sensitivity by ROS-mediated autophagic flux in human breast cancer cells
Source: BMC Cell Biol. 2017 Jul 20;18:26. doi: 10.1186/s12860-017-0141-0 (PMC5520384; doi:10.1186/s12860-017-0141-0)
Supplement: Additional file 1: — Figure S1. 1H–NMR of Cladosporol A. Figure S2. 13C–NMR of Cladosporol A. Figure S3. HPLC spectra of Cladosporol A. (DOC 374 kb) [file 12860_2017_141_MOESM1_ESM.doc]

**Supporting Information**

# Cladosporol A triggers apoptosis sensitivity by ROS-mediated autophagic flux in human breast cancer cells

Authors: Mytre Koul^1,4^, Ashok Kumar^1,4^, Ramesh Deshidi^2,4^, Vishal Sharma^3,4^, Rachna D Singh^1,4^, Jasvinder Singh^1,4^, Parduman Raj Sharma^1,4^, Bhahwal Ali Shah^2,4^, Sundeep Jaglan^3,4^, Shashank Singh^1,4*^

1. Cancer Pharmacology Division, CSIR-Indian Institute of Integrative Medicine, Jammu

2. Natural Product Chemistry, CSIR-Indian Institute of Integrative Medicine, Jammu

3. Microbial Biotechnology Division, CSIR-Indian Institute of Integrative Medicine, Jammu

4. Academy of Scientific & Innovative Research (AcSIR), CSIR, New Delhi

**List of Supporting Information**

**Page**

Figure 1S. ^1^H-NMR of Cladosporol A 3

Figure 2S. ^13^C-NMR of Cladosporol A 4

Figure 3S. HPLC spectra of Cladosporol A 5

**Cladosporol A (HPLC purified > 98%)**

^1^H NMR (400 MHz, CDCl_3_) δ 12.59 (d, J = 6.7 Hz, 1H), 8.62 (s, 1H), 7.33 – 7.20 (m, 2H), 7.01 (t, J = 15.8 Hz, 1H), 6.95 (d, J = 8.6 Hz, 1H), 6.80 (d, J = 8.3 Hz, 1H), 6.23 (d, J = 7.6 Hz, 1H), 5.43 (d, J = 8.7 Hz, 1H), 4.88 (dd, J = 8.2, 4.8 Hz, 1H), 4.10 – 4.04 (m, 1H), 3.87 (d, J = 4.5 Hz, 1H), 3.53 (t, J = 14.0 Hz, 1H), 2.76 (t, J = 6.6 Hz, 2H), 2.51 (dt, J = 18.7, 5.5 Hz, 1H), 2.21 (dt, J = 22.4, 8.2 Hz, 1H); ^13^C NMR (125 MHz, CDCl_3_) δ 205.3, 194.9, 162.7, 137.3, 136.4, 132.2, 122.5(d, J = 7.4 Hz), 120.0, 115.8, 67.5, 56.1, 55.2, 40.1, 36.8, 31.0. HRMS (ESI^+^) calculated for C_20_H_16_O_6_ [M+H]^+^: 353.1020, found: 353.1015.

Fig. 1S  ^1^H-NMR data of compound Cladosporol A

Fig. 2S ^13^C--NMR data of compound Cladosporol A

**
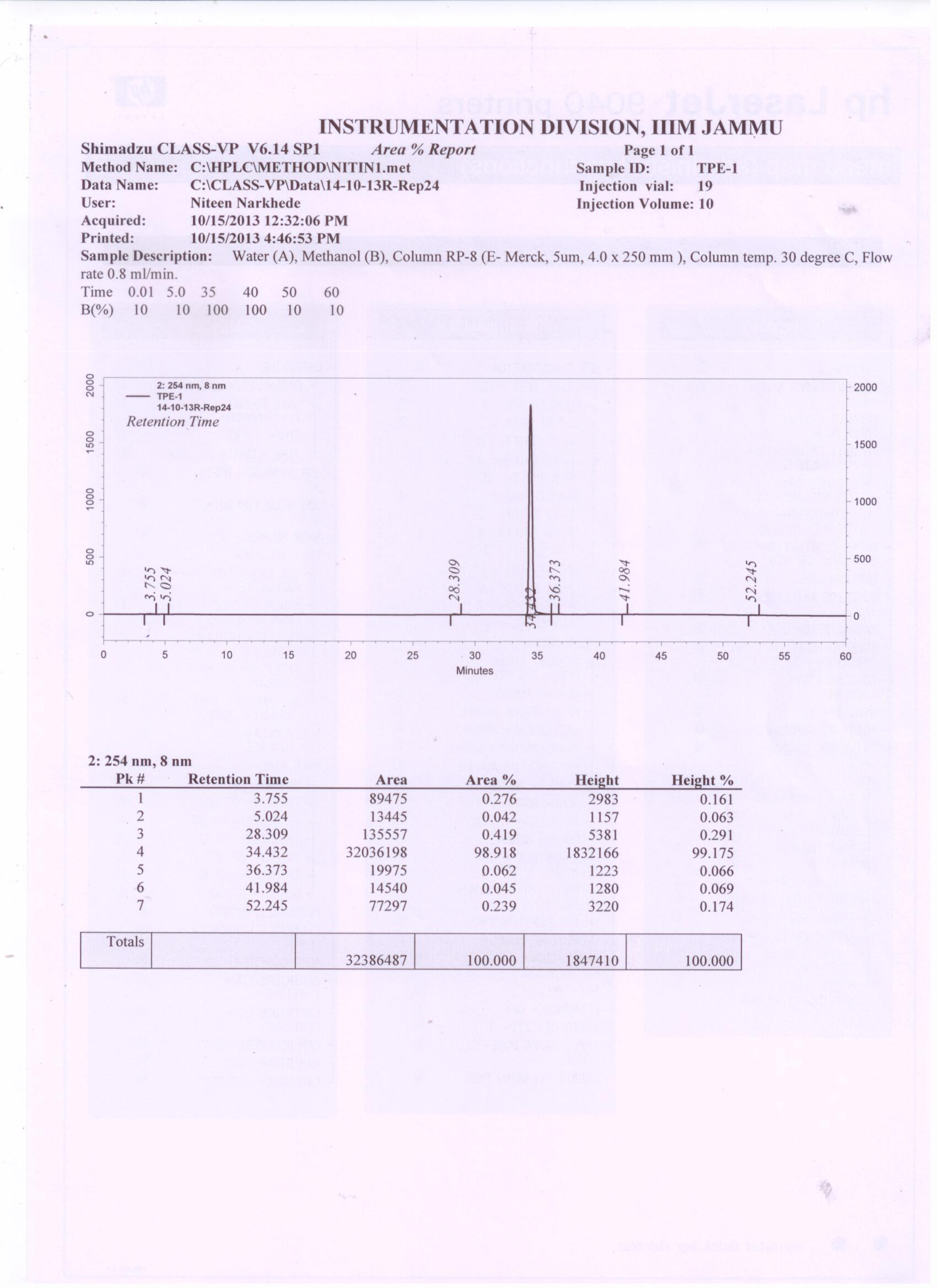
**

Fig. 3S HPLC spectra of Cladosporol A
